# Clinical Assessment and Management of Acute Spinal Cord Injury

**DOI:** 10.3390/jcm13195719

**Published:** 2024-09-25

**Authors:** Christian Quinones, John Preston Wilson, Deepak Kumbhare, Bharat Guthikonda, Stanley Hoang

**Affiliations:** Department of Neurosurgery, Louisiana State University Health Shreveport, Shreveport, LA 71103, USA; cjq001@lsuhs.edu (C.Q.); jpw002@lsuhs.edu (J.P.W.J.); deepak.kumbhare@lsuhs.edu (D.K.); bharat.guthikonda@lsuhs.edu (B.G.)

**Keywords:** spinal cord injury, acute management, complications

## Abstract

The information contained in this article is suitable for clinicians practicing in the United States desiring a general overview of the assessment and management of spinal cord injury (SCI), focusing on initial care, assessment, acute management, complications, prognostication, and future research directions. SCI presents significant challenges, affecting patients physically, emotionally, and financially, with variable recovery outcomes ranging from full functionality to lifelong dependence on caregivers. Initial care aims to minimize secondary injury through thorough neurological evaluations and imaging studies to assess the severity of the injury. Acute management prioritizes stabilizing respiratory and cardiovascular functions and maintaining proper spinal cord perfusion. Patients with unstable or progressive neurological decline benefit from timely surgical intervention to optimize neurological recovery. Subacute management focuses on addressing common complications affecting the respiratory, gastrointestinal, and genitourinary systems, emphasizing a holistic, multidisciplinary approach. Prognostication is currently based on neurological assessments and imaging findings, but emerging biomarkers offer the potential to refine outcome predictions further. Additionally, novel therapeutic interventions, such as hypothermia therapy and neuroprotective medications are being explored to mitigate secondary damage and enhance recovery. This paper serves as a high-yield refresher for clinicians for the assessment and management of acute spinal cord injury during index admission.

## 1. Introduction

SCI poses a significant challenge, impacting patients physically, emotionally, and financially. Individuals with SCI often face a broad spectrum of difficulties, from varying degrees of physical disability to severe psychosocial disturbances. The variability in recovery further complicates the situation, with some patients regaining full functionality and independence while others remain dependent upon caregivers. Understanding the multifaceted nature of SCI will aid clinicians in delivering comprehensive care and facilitate the creation of specialized support systems for affected individuals. This paper discusses the nature of SCI, emphasizing the importance of initial care and assessment to minimize secondary injury. Key aspects in the assessment and management of SCI include emergency management, thorough neurological evaluations using standardized assessments, imaging for prognostication, and surgical planning. Acute management focuses on addressing common initial complications. In patients with spinal cord compression, timely surgical intervention is key to optimizing neurological recovery. Prognostication relies on initial assessments, magnetic resonance imaging findings, and emerging biomarkers.

Globally, the incidence of SCI has increased from 1990 to 2019, and this trend was expected to continue through 2023, along with an increase in life expectancies for individuals with SCI [1]. The National Spinal Cord Injury Statistical Center reports approximately 18,000 new traumatic SCI cases annually in the U.S., with an average patient age of 43 years. Around 60% of SCIs result from falls and vehicle accidents, 15.4% from violence, and 79% occur in males, predominantly within the non-Hispanic White population (55.5%) [2]. This publication will focus on traumatic SCIs, as over 75% of cases are due to traumatic events.

In addition to the challenges of physical recovery, the financial impact of SCI has been well documented in the literature to date, with the National Spinal Cord Injury Statistical Center estimating the first year of medical cost after SCI to be USD 1,369,755, with USD 237,852 incurred each subsequent year for ongoing injury management [2]. Significant psychosocial disturbances have been described in patients with SCI. Studies have shown that certain subpopulations are at particular risk of these complications. One study found significantly increased rates of depression in female SCI tetraplegics, those reporting lower education levels, histories of suicidal ideation or attempts, and patients whose primary caregiver was not a spouse or parent [3]. Other comorbidities, such as an increase in alcohol dependence, follow a similar trend, with an increased rate of binge drinking in SCI patients during the first 17 months after injury [4].

The complexities of SCI extend beyond immediate physical impairments, encompassing emotional and financial burdens that negatively impact these patients’ daily lives. The significant medical costs, persistent pain, increased depression rates, and substance use seen with these patients highlight the challenges faced by these individuals and society as a whole. Addressing these issues requires a holistic, multidisciplinary approach that considers both the physical and psychosocial aspects of SCI, ensuring that patients receive the necessary support to improve and maintain their quality of life. 

## 2. Initial Care and Assessment

The assessment and care of SCI entails thorough initial care and skilled utilization of a standardized evaluation. Becoming proficient in the basics of initial care and assessment will aid healthcare workers in delivering the best care to patients with SCI. The following sections provide an overview of the pathophysiology and describe the critical steps in emergency management and stabilization. Standardized neurological assessments are discussed as well as how imaging aids in prognostication. Finally, future directions for SCI assessment are discussed.

The mechanisms by which the spinal cord is damaged can be divided into two phases: primary and secondary injury. Primary injury refers to the initial mechanical or otherwise destructive force causing damage to the spinal cord [5]. Secondary injuries can be directly or indirectly caused by primary injuries and have been attributed to pathologic vascular disruption, electrolyte imbalance, and immunological dysregulation in the setting of cell injury and mitochondrial function [6]. In the initial care and management of patients with acute SCI, it is paramount to minimize secondary injuries.

The management of SCI begins immediately after a primary injury. In the case of traumatic SCI, emergency medical personnel are guided by the Advanced Trauma and Life Support (ATLS) guidelines that prioritize airway, breathing, and circulation [7]. Patients suspected of having an SCI should be immobilized with a cervical collar or placed on a backboard [8]. Prompt transportation to a medical facility with neurosurgical expertise should ensue, and upon arrival, patients are assessed for other life-threatening injuries. After initial stabilization, a thorough assessment (with particular attention to the history and mechanism of injury) and a neurological exam should be performed. Abnormal neurological exams or initial imaging suggestive of SCI should be further investigated with advanced cross-sectional imaging.

### Neurological Assessment in SCI

The American Spinal Injury Association (ASIA) developed the International Standards for Neurological Classification of Spinal Cord Injury (ISNCSCI), which serves as the standardized method for evaluating SCIs [9]. The ASIA Impairment Scale (AIS) is a key component of this evaluation, used to grade the severity of spinal cord injuries based on sensory and motor functions. This grading system helps clinicians identify the level and completeness of the injury, supporting patient management decisions. Other standalone assessments commonly used for patients with SCI include the Spinal Cord Independence Measure (SCIM), which evaluates a patient’s functional independence by examining their ability to perform daily living activities [10], and the Walking Index for Spinal Cord Injury (WISCI), which measures the level of assistance required for walking after SCI [11]. The SCIM offers a comprehensive assessment of SCI on a patient’s overall independence by assessing three broad areas: self-care, respiration and sphincter management, and mobility. The WISCI, in contrast, more specifically evaluates a patient’s ability to ambulate a 10 m distance. This 21-level scale describes ambulatory status from fully independent without the use of assistance, assistant devices, or braces to an inability to stand or walk. Both the SCIM and WISCI aid clinicians in setting rehabilitation goals and monitoring changes in mobility and functional independence. This review will discuss the comprehensive examination described by the ISNCSCI, as proposed by ASIA, for initial assessment after acute SCI.

The AIS exam evaluates both sensory and motor functions across dermatomes and myotomes, which correspond to the skin segments and muscle groups innervated by specific spinal levels [9]. The latter two terms allow clinicians to designate an AIS (A–E). Sensory assessment involves testing two modalities—light touch and sharp–dull discrimination (pin prick)—on both sides of the patient’s body [9]. Light touch is tested using a cotton thread, while pin-prick assessment uses a safety pin or an 18-gauge needle, requiring the evaluation of both the sharp and dull ends. Each of the 28 dermatomes is assessed for both light touch and pin-prick sensation. The S4 and S5 dermatomes require a rectal exam to evaluate pressure sensation, and the patient is also asked to gently squeeze their sphincter. Individual dermatome sensory scores (Table 1) range from 0 (absent sensation) to 2 (normal sensation compared to the cheek). The total sensory score is the sum of both sensory modalities across all dermatomes [9].

The motor exam involves assessing 10 myotomes on both sides of the body [9]. Each myotome is evaluated and given a score (Table 2), with the total motor score being the sum of the scores from all myotomes.

The sensory level is defined as the most caudal dermatome where both sensory modalities are normal, while the motor level is the most caudal level with a muscle strength score of three or greater [9]. These levels help determine the completeness of the injury.

**Figure 1:**
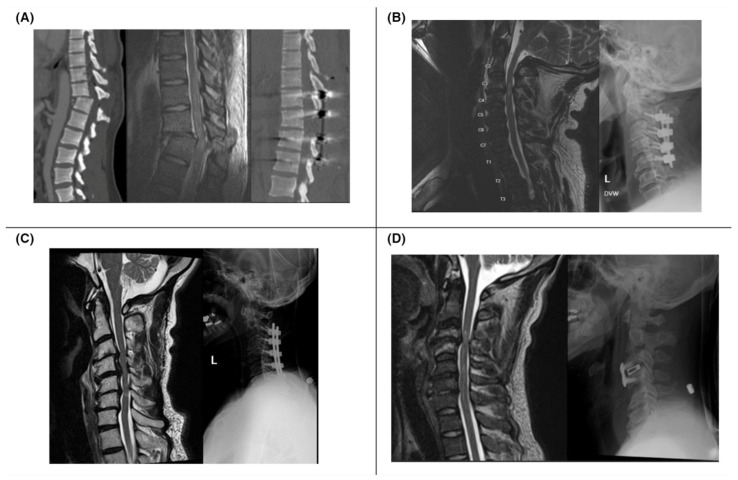
(**A**) Patient with lower extremity motor and sensory loss after MVC (ASIA A); CT (left) and sagittal T2 MRI (middle) show T11–12 fracture dislocation; postoperative CT shows improved alignment (right). (**B**) Patient with intact sensation but no motor function after fall (ASIA B); preoperative MRI (left) shows C3–4 stenoses and cord contusion; postoperative decompression and fusion from C2–5 X-ray (right). (**C**) Patient with 2/5 hand weakness and intact sensation (ASIA C) after fall; MRI with spinal cord contusion at C4–6 (left); postoperative X-ray with instrumented decompression and fusion (right). (**D**) Patient with hand paresthesia and intact motor function after fall (ASIA D); MRI with traumatic disc herniation at C3–4 (left); postoperative C3–4 anterior cervical discectomy and fusion X-ray (right).

A complete SCI is characterized by the loss of both sensory and motor functions below the neurologic level of injury (NLI), while an incomplete injury involves preservation of either sensory or motor function below the level of injury [9]. Based on these assessments, an AIS grade (A–E) is assigned (Table 3) to classify the severity of the injury. Figure 1 provides examples of pre- and post-operative imaging of SCI patients with varying AIS grades.

## 3. Acute Management

### 3.1. Respiratory Function and Cardiovascular Stability

Monitoring respiratory function in patients with cervical SCI is imperative to mitigate severe respiratory risks. These patients are particularly vulnerable due to the potential disruption of innervation to the diaphragm (C3–C4), intercostal muscles (T1–T12), and abdominal muscles (T12–L1) [12]. In a study by Velmahos et al., 95% of patients with complete quadriplegia and an injury level above C5 required intubation [13].

Close hemodynamic monitoring is essential in the acute management of SCI to ensure adequate spinal cord perfusion and prevent secondary injury. Maintaining spinal cord perfusion pressure improved neurological outcome in patients with traumatic SCI [12,14]. Close blood pressure control to optimize spinal cord perfusion pressure is best achieved by invasive means [15]. Disruption of the sympathetic outflow from the spinal cord can compromise a patient’s cardiovascular and circulatory systems. If an SCI is at or above the level of T1–6 [16], the sympathetic cardiac function can be disrupted [17]. This disruption most commonly manifests as bradycardia [17]. Lack of sympathetic input to the large blood vessels can cause pooling of blood and decreased venous return [6]. Decreased sympathetic drive can lead to a combination of hypotension and bradycardia, which is termed neurogenic shock. Patients with neurogenic shock should be resuscitated with crystalloid solutions such as lactate ringer or normal saline [17]. In the case of neurogenic shock that is refractory to fluid resuscitation, vasopressors or parasympathetic antagonists should be considered [18]. Due to these hemodynamic alterations, care should be taken to administer agents that preserve hemodynamic stability, as some commonly used medications such as propofol and thiopental may cause hypotension [17]. Through vigilant monitoring and appropriate interventions, healthcare providers can support cardiovascular stability and enhance neurological outcomes for SCI patients.

### 3.2. Pharmacological Considerations: Methylprednisolone

Methylprednisolone is a glucocorticoid whose use in the treatment of acute SCI has been a topic of significant research and debate over the years. Initial studies in cats found that methylprednisolone decreased the degree of secondary SCI [19]. In the National Acute Spinal Cord Injury Study (NASCIS I) trial, methylprednisolone sodium succinate (MPSS) was shown to provide patients with a degree of therapeutic benefit after SCI [20]. However, the subsequent NASCIS II trial of methylprednisolone versus naloxone in acute SCI was not found to improve patient outcomes [21]. Two major criticisms of MPSS administration are that it may delay wound healing and increase the risk of infection. An analysis of the NASCIS II and III studies claimed that high-dose methylprednisolone after acute SCI was not associated with an increased risk of steroid-induced side effects [22]. A systematic review of steroid administration in SCI rated the strength of recommendation for the use of MPSS in adults with acute spinal cord injury as “weak” [23]. Despite extensive research, no definitive evidence supports the routine use of MPSS in acute SCI management.

## 4. Surgical Management

The primary objective of surgical intervention in SCI is to decompress and stabilize the spine, as to limit secondary injury and achieve mechanical stability [24]. In cases of mechanical impingement on the spinal cord, surgical decompression can restore blood flow [25]. This emphasizes the importance of thorough imaging and clinical evaluation, particularly given the absence of standardized guidelines for surgical timing and the optimal surgical approach, aside from the general principle of “time is spine” [26]. The World Society of Emergency Surgery and the European Association of Neurological Societies described 17 variables to optimize SCI patients prior to surgery [27]. These recommendations should be applied in a timely manner to avoid the delay of surgical decompression.

An important aspect of managing SCI is the assessment of spinal stability, which is defined as the loss of structural integrity that compromises the spine’s ability to protect the spinal cord or nerve roots [28]. The spinal cord is protected by vertebrae interconnected by ligaments, including the anterior and posterior longitudinal ligaments, which maintain the patency of the spinal canal during daily movements. In the context of SCI caused by vertebral fractures or dislocations, the structural integrity of the spine is compromised, and further movement could exacerbate spinal cord injury. The presence of dislocations is associated with increased neurologic deterioration and is typically treated with spinal decompression and stabilization [24,29,30]. Imaging studies are critical in assessing spinal stability, with certain fracture patterns, mechanisms, and injury types offering insights into stability. Notably, SCI can occur without bony fractures, underscoring the importance of MRI in such evaluations [31]. Surgical decompression within 24 h has been shown to improve neurologic outcomes, as demonstrated by the STASCIS Trial [32].

When considering surgical intervention, factors such as stability, severity, and fracture morphology must be evaluated. Various classification systems of spinal injury exist, which are based on anatomical relationships or injury mechanisms. One of the earliest systems, developed by Denis in 1983, categorized thoracolumbar injuries by their impact on the anterior, middle, or posterior column of the spine [33]. However, this system does not provide guidance on treating unstable injuries [30]. In 2005, the Thoracolumbar Injury Classification and Severity Score (TLICS) devised a scoring system based on morphology, neurological involvement, and posterior ligament integrity that described the nature of injury and provided guidance on the need for surgical intervention [34]. Similar to the TLICS, the Subaxial Cervical Spine Injury Classification System uses injury morphology, ligamentous integrity, and the patient’s neurological status to describe the cervical spine [35].

Surgical approaches vary based on the pathology. For instance, an SCI caused by an anteriorly displaced cervical disc is typically managed with an anterior approach [36] while a patient with central cord syndrome due to multi-level compression is managed with a posterior approach [37]. Preparing a patient for surgical intervention can present challenges both before and after admission. A study of the National Trauma Data Bank reported that most delays to surgical interventions for SCI occurred after admission, with the majority of patients not receiving surgery within 24 h of injury [38]. Battistuzzo et al. found the average time from admission to the operating room to decompress cervical SCI was 12.5 h [39].

## 5. Management of Subacute Complications in SCI

Following an SCI, early management often necessitates acute respiratory and cardiopulmonary support. Mortality rates are notably high during the first and second years post-injury, with a reported 71% mortality among patients with high cervical injuries between 1990 and 2004 [40]. In the subacute phase, complications such as dysphagia, respiratory issues, and pressure ulcers are prevalent. The frequent occurrence of these complications contributing to premature mortality underscores the critical need for prompt identification and effective intervention to improve outcomes and decrease the likelihood of rehospitalization [41].

Respiratory complications are particularly prevalent in patients with high cervical spine injuries, especially if the normal functioning of the intercostal and abdominal muscles is compromised [42]. These complications arise due to the impaired ability to perform maximal inspiration, expiration, and an effective cough reflex, which are critical for clearing airway secretions. Consequently, patients are at an increased risk of aspiration, mucus plugging, and impaired gas exchange, which can lead to pneumonia [43]. The severity of these respiratory issues is well-documented, with studies indicating that respiratory complications are the most common cause of hospitalization in tetraplegic patients [42]. Given the high incidence of dysphagia in patients with high SCI, particularly in patients with tetraplegia, this subacute complication increases the risk of poor oxygenation and aspiration pneumonia [44]. The incidence of dysphagia in this population is notable; Shem et al. reported a 40% occurrence rate in tetraplegic patients [45], while Wolf and Meiners found that 21 out of 50 acute SCI patients with dysphagia experienced an aspiration event [46]. The onset of dysphagia in SCI patients is often attributed to inflammation and swelling at the site of vertebral fractures or as a consequence of surgical intervention [44].

The management of skin integrity is closely linked to overall mobility and quality of life in patients with SCI. These factors are further influenced by the control of bladder and bowel functions, which exemplifies the importance of comprehensive patient care. Pressure ulcers and urinary tract infections (UTIs) are potentially life-threatening complications following SCI that have been found to extend patient hospital stays [47]. Chronic pressure ulcers have been identified as the strongest predictor of mortality in SCI patients [48] and are more prevalent among paraplegics [42]. The management of pressure ulcers involves scheduled patient repositioning, limiting skin exposure to moist environments, and maintaining the head of the bed at the lowest feasible level [49].

The extent of SCI affects sympathetic, parasympathetic, and somatic innervation to varying degrees, leading to potential disturbances in bladder and bowel functions, such as retention or incontinence. These disruptions arise because normal bladder and bowel function depend on the coordinated actions of these nervous systems: the sympathetic system relaxes the detrusor muscle and contracts the internal urinary sphincter, while the parasympathetic system promotes bladder contraction and relaxation of the internal sphincter, both of which are essential for effective bladder emptying [50,51].

UTIs are a leading cause of rehospitalization in SCI patients [42]. The management of bladder dysfunction includes monitoring fluid intake and output, conducting timed and post-void residual bladder scans, and preferring clean intermittent catheterization over indwelling Foley catheters. A proactive approach to UTI management, including antibiotic therapy and urine cultures when indicated, is vital to reducing the morbidity of these complications [5]. The same disruptions in autonomic regulation that affect the urinary system can also impact gastrointestinal function [52]. The sympathetic nervous system aids in stool retention, while the parasympathetic system promotes peristalsis and facilitates defecation. A disruption of these systems due to SCI can result in either constipation or incontinence of stool. In cases of constipation, the recommended management includes proper hydration, a diet high in fiber, and the use of oral or rectal laxatives and enemas [53]. For patients experiencing incontinence, regular assessment of linens is necessary to prevent skin breakdown from moisture, which increases the risk of pressure ulcers. These effects on genitourinary and gastrointestinal functions underscore the importance of personalized care for each SCI patient, tailored to the specific extent and level of their injuries.

Effective pain management is essential for patients with SCI to optimize comfort and facilitate participation in physical rehabilitation. Pain in SCI patients typically falls into two main categories: nociceptive pain, which includes musculoskeletal and visceral pain, and neuropathic pain [54]. A 2019 review on pain management in SCI recommended the use of the World Health Organization’s Analgesic Ladder as a structured approach to pain control. This ladder suggests starting with oral non-opioid analgesics, such as acetaminophen, along with adjuvant therapies, and progressing to mild and then stronger opioids as needed [55]. Adjuvant therapies, which may include anticonvulsants, corticosteroids, and anxiolytics, play a key role in managing specific pain types, particularly neuropathic pain.

## 6. Prognostic Factors and Future Directions in SCI Management

The initial assessment of patients with SCI, including history, physical examination, and imaging, is fundamental in predicting the clinical course and guiding management. High-energy mechanisms of injury are associated with a higher likelihood of complete motor SCI, highlighting the importance of thorough evaluation to tailor immediate and long-term care [56].

### 6.1. Initial Assessment and Decision-Making Tools

Accurate initial assessment is essential in SCI management, particularly in identifying the need for imaging and intervention. Clinical decision-making tools, such as the NEXUS criteria, play a significant role in assessing trauma patients with low suspicion of cervical spine injury. The NEXUS criteria allow clinicians to forgo spine imaging when all of the following conditions are met: absence of midline cervical tenderness, no intoxication or altered mental status, no neurological deficit, and no distracting injuries [57]. This approach helps reduce unnecessary imaging and focuses resources on patients with higher clinical indications for imaging.

### 6.2. Imaging Modalities for SCI Prognostication

Imaging is a key component in determining the level and extent of SCI, which assists with surgical planning and predicting neurological outcomes. CT scans are typically preferred for initial imaging due to their high sensitivity for detecting bony injuries and rapid acquisition time, though they do not visualize soft tissues or spinal cord structures [58]. MRI, superior for identifying soft tissue injuries such as ligamentous damage and spinal cord edema, is the gold standard for visualizing SCI [58,59]. High-risk imaging criteria include factors such as altered mental status, significant head injuries, age over 65, and extremity paresthesia [60]. The combination of AIS and MRI provides a mechanism for prognostication after traumatic SCI [61]. MRI, particularly sagittal T2 sequences, provides valuable insight into the level and severity of the injury, correlating with potential neurological outcomes [62]. The AIS, recognized for its simplicity and reproducibility, helps categorize the extent of motor and sensory deficits, aiding in predicting recovery trajectories [9]. Advanced MRI techniques, such as diffusion tensor imaging, are being explored for their ability to detect subtle changes in white and gray matter, further refining prognostic accuracy in chronic SCI [63,64].

In cases of Spinal Cord Injury Without Radiographic Abnormality (SCIWORA), clinical observation and judgment are paramount. A case report highlighting the importance of vigilance, described a patient who developed paraplegia several hours post-injury despite an initially benign physical exam and negative imaging findings [65]. This underscores the need for careful monitoring when radiological results do not align with clinical presentation.

## 7. Emerging Prognostic and Therapeutic Candidates in SCI Management

Advancements in SCI management may increasingly rely on integrating biomarkers to refine initial assessments and improve prognostication. Various studies have identified potential biomarkers, including hematological and cerebrospinal fluid (CSF) markers, which offer promise in predicting SCI outcomes. For example, combining MRI with CSF biomarkers, such as neuron-specific enolase (NSE) and the protein S100B, enhances the accuracy of predicting injury severity and patient prognosis [66]. Proteomic CSF analysis in animal models has identified 12 protein biomarkers that could serve as indicators of SCI severity and recovery, highlighting their potential clinical utility (Table 4) [67]. However, larger studies are needed to validate these findings and establish these biomarkers as reliable tools in clinical practice [68].

Research into microRNAs and specific pathways, such as the Rho pathway, could also contribute to more effective diagnostics and therapeutic strategies for SCI [67,72]. Interleukins (ILs), particularly IL-6 and IL-10, play significant roles in regulating inflammation and immune responses after SCI, making them valuable for assessing injury severity and guiding treatment [67,72]. Moreover, neuropeptide FF has been proposed as a potential marker for predicting chronic complications, such as cognitive decline in SCI patients [74].

### 7.1. Novel Therapeutic Strategies

The primary objective in SCI treatment is to minimize secondary injury, as the initial damage from the primary insult is irreversible. This has led to a focus on protective therapies (Table 5), including molecular, cellular, and electrical stimulation approaches, aimed at improving patient outcomes [75]. Riluzole, a neuroprotective drug that blocks sodium channels and inhibits glutamate release, has shown mixed results in clinical trials, underscoring the need for further investigation [25,76,77]. Another novel drug is minocycline—an antibiotic with anti-apoptotic properties hypothesized to reduce cell death after SCI [78]. Other emerging therapies, such as Ganglioside Monosialotetrahexosylganglioside (GM-1) and mouse Nerve Growth Factor (mNGF), function via neuroprotective mechanisms [79]. Additionally, other studies have identified immune target genes (TMEM176A and TMEM176B) whose protein products prevent nervous system recovery [80]. Hypothermic therapy, which involves reducing the body’s metabolic rate to protect nerves and organs, has also emerged as a promising approach [81,82,83,84]. Innovations such as artificial hibernation are being explored to reduce side effects of SCI; however, more extensive studies are required to confirm these benefits [82,83]. Regenerative therapies, such as the use of mesenchymal and neural stem cells, offer potential for repairing spinal cord damage, though their efficacy needs further validation [85]. Inhibiting the Rho pathway with agents like BA-210 may also promote recovery by targeting growth-inhibitory proteins after CNS injury [72].

### 7.2. Advances in Motor Function Assessment and Rehabilitation

Neurostimulation therapies (Table 6), including spinal cord stimulation, transcranial magnetic stimulation (TMS), and epidural electrical stimulation (EES), show promise in restoring function and managing complications such as urinary incontinence by activating residual neurons and supporting nerve repair [85]. These approaches have demonstrated significant potential in enhancing motor functions, including standing and walking independently, and are critical in assessing recovery trajectories in SCI patients [87]. Techniques such as electromyography (EMG) and TMS are not only used to evaluate motor function but also track recovery progress, leading to the development of predictive tools like the “trunk control test”, which forecasts independence and ambulation one year after injury [88,89,90,91].

However, neurostimulation technologies face challenges, including high costs, technical complexity, and current variable clinical outcomes, highlighting the need for ongoing research to optimize their use in clinical settings. Additionally, novel therapeutic approaches, such as minocycline—an antibiotic with anti-apoptotic properties—are being investigated for their potential to reduce cell death post-SCI, reflecting the broad range of therapeutic strategies under exploration [78]. Hodel et al. emphasized the need for continued research to refine these predictive models and integrate advanced therapies, such as stem cell treatments and neurostimulation, into standard care for SCI patients [97].

## 8. Knowledge Gaps and Future Directions in SCI Research

The current landscape of SCI research would benefit from advancements in several areas to improve clinical outcomes and patient quality of life. A review of clinical trials in SCI identified the necessity for refined methodologies, well-defined endpoints, and standardized inclusion and exclusion criteria to enhance the reliability and applicability of research findings [98]. Future research should prioritize factors directly impacting functional recovery, such as hand function and bladder control, often overlooked in acute SCI management [99]. Advancements in imaging technologies and novel biomarkers offer opportunities to refine prognostication and tailor treatment strategies [61]. Additionally, the timing and impact of surgical interventions remain uncertain; precise evidence-based guidelines are needed to inform decision-making regarding the optimal timing for surgery [100]. The variability in surgical approaches and the lack of robust guidelines for intraoperative neuromonitoring underscore the need for standardization. Establishing postoperative care protocols, particularly for maintaining adequate spinal cord perfusion, could significantly improve neurological outcomes and decisions [101]. Addressing complications such as neuropathic pain, autonomic dysfunction, and spasticity is crucial for enhancing post-discharge quality of life. Developing strategies to mitigate these secondary injuries should be a research priority.

To close these knowledge gaps, future studies must focus on creating evidence-based guidelines that standardize treatment protocols across all aspects of SCI care, from surgical interventions to long-term management. This approach will not only advance scientific understanding but also lead to tangible improvements in patient care throughout the continuum of SCI treatment.

## Figures and Tables

**Table 1:** Sensory score.

Score	Finding
0	Absent (no sensation)
1	Abnormally decreased or increased
2	Normal (as compared to sensation on cheek)
Not Testable	Due to other injuries or baseline deficit

**Table 2:** Motor score.

Score	Finding
0	Total paralysis
1	Muscle contraction (can be felt or visualized)
2	Can move myotome in a plane parallel to gravity
3	Can move myotome against gravity and mild resistance
4	Can move myotome against gravity and moderate resistance
5	Can move myotome against gravity and full resistance

**Table 3:** ASIA Impairment Scale (AIS).

Grade	Features
A	Complete injury. No sensory or motor function below the NLI
B	Incomplete injury. Only sensory function below the NLI
C	Incomplete injury. Motor strength < 3 and sensory function below the NLI
D	Incomplete injury. Motor strength ≥ 3 and sensory function below the NLI
E	Normal exam

**Table 4:** Biomarkers for prognostication in spinal cord injury.

Biomarker	Mechanism of Action/Description
Interleukin-6, 10 [69]	IL-6 promotes inflammation and hinders remyelination IL-10 has anti-inflammatory effects that regulate the immune response
Neuropeptide FF [70]	Predicts cognitive decline in previously healthy patients after acute SCI
Liver Function Tests [70]	Proposed as prognostic biomarkers for SCI outcomes
RNA Biomarkers [71]	RNA biomarkers reflect gene expression changes during states of inflammation, cell death, and tissue repair, with potential for monitoring SCI progression
Proteomic CSF Analysis [67]	Identified 12 potential protein biomarkers in rat models; may aid in diagnosis and prognosis or as therapeutic targets
Rho Pathway [72]	Controls neuronal responses to growth-inhibitory proteins post-CNS injury, relevant for SCI
Neuron-Specific Enolase (NSE) and S100B [73]	NSE and S100B are associated with neuronal damage and glial activation; elevated levels in CSF indicate injury severity and ongoing neurological impairment

**Table 5:** Emerging interventions and therapies for spinal cord injury.

Intervention/Therapy	Mechanism of Action
Hypothermia [81,82,83,84]	Reduces metabolic rate, potentially decreasing secondary injury by lowering body temperature
GM-1 + mNGF [79]	Reduce excitatory neurotransmitters, Na^+^-K^+^-ATPase and Ca^2+^-ATPase activity, inflammation, oxidative stress, calcium buildup, and apoptosis; promote nerve growth and membrane repair
BA-210 [72]	Inhibits the Rho pathway, which is involved in growth-inhibitory proteins after CNS injury, promoting recovery
Riluzole [86]	Blocks voltage-sensitive sodium channels and inhibits presynaptic calcium-dependent glutamate release, providing neuroprotective effects
Immune Therapy Targets [80]	Target transmembrane proteins on immature dendritic cells, preventing their maturation, which may aid in SCI recovery
Minocycline [78]	Acts as an antibiotic with anti-apoptotic properties, potentially reducing cell death post-SCI

GM-1: Ganglioside Monosialotetrahexosylganglioside; mNGF: mouse Nerve Growth Factor.

**Table 6:** Neurostimulation therapies and motor function assessment in spinal cord injury.

Therapy/Assessment	Mechanism of Action/Therapeutic Effects
Transcranial Stimulation [92]	Visual illusions reduced neuropathic pain in SCI patients
Transcutaneous Electrical Stimulation [93]	Combined with a drug, it enabled voluntary motor function in paralyzed patients, possibly by reactivating dormant motor pathways
Transcutaneous Electrical Nerve Stimulation (TENS) and Functional Electrical Stimulation (FES) [94]	Both provided similar anti-spasticity effects and are potential therapeutic options
Electrical Stimulation for Neurogenic Bowel [95]	A systematic review found insufficient evidence for the efficacy of transcutaneous, transrectal, sacral nerve, or intravesical stimulation in treating neurogenic bowel dysfunction
Transcranial Magnetic Stimulation (TMS) [96]	Shown to improve motor function performance in SCI patients
Electromyography (EMG) + TMS [88,89,90]	Used to track motor function recovery and evaluate abdominal muscle integrity, leading to the development of the “trunk control test”, predictive of independence and ambulation
